# Universal approach to actigraphic sleep/wake scoring, verified against 5 classic algorithms on 3 datasets.

**DOI:** 10.1038/s41598-026-45568-0

**Published:** 2026-04-17

**Authors:** Piotr Biegański, Anna Duszyk-Bogorodzka, Dorota Wołyńczyk-Gmaj, Batłomiej Gmaj, Piotr Durka

**Affiliations:** 1 Faculty of Physics, Biomedical Physics Division, University of Warsaw, 5 Pasteura st., 02-093 Warsaw, Poland; 2 Institute of Psychology, Behavioural Neuroscience Lab, SWPS University, 19/31 Chodakowska st., 03-815 Warsaw, Poland; 3 Department of Psychiatry, Medical University of Warsaw, 27 Nowowiejska st., 00-665 Warsaw, Poland

**Keywords:** Computational biology and bioinformatics, Engineering, Health care, Mathematics and computing

## Abstract

Actigraphy is a non-invasive and inexpensive method to monitor sleep/wake patterns in a natural environment via a wrist-worn activity sensor. Traditionally, detection of sleep/wake periods from actigraphic data relies on smoothing and thresholding the time series of recorded “activity counts”. The first step is implemented by convolution with empirically chosen coefficients, tailored separately for the data and hardware used in each study. We propose to implement this step via a universal low-pass filter, applicable to wide ranges of recording hardware and sampling rates. For verification of this approach, we used 1635 overnight coregistrations of actigraphic and polysomnographic (PSG) data from three different datasets, including one dataset recorded for this study. Optimizations of the filter for concordance of sleep/wake scoring with PSG for different subsets of these data converged to similar parameters, which we tentatively treat as fluctuations around the characteristics of a universal filter. We assess the performance of the proposed approach and five classic algorithms (Cole-Kripke, Sazonov, Scripps, UCSD and Webster) in the same cross-validation scheme. Concordance with PSG, achieved using the universal filter, is significantly higher (at $p < 0.001$) than any of the classical algorithms for the most relevant metrics.

## Introduction

**Fig. 1:**
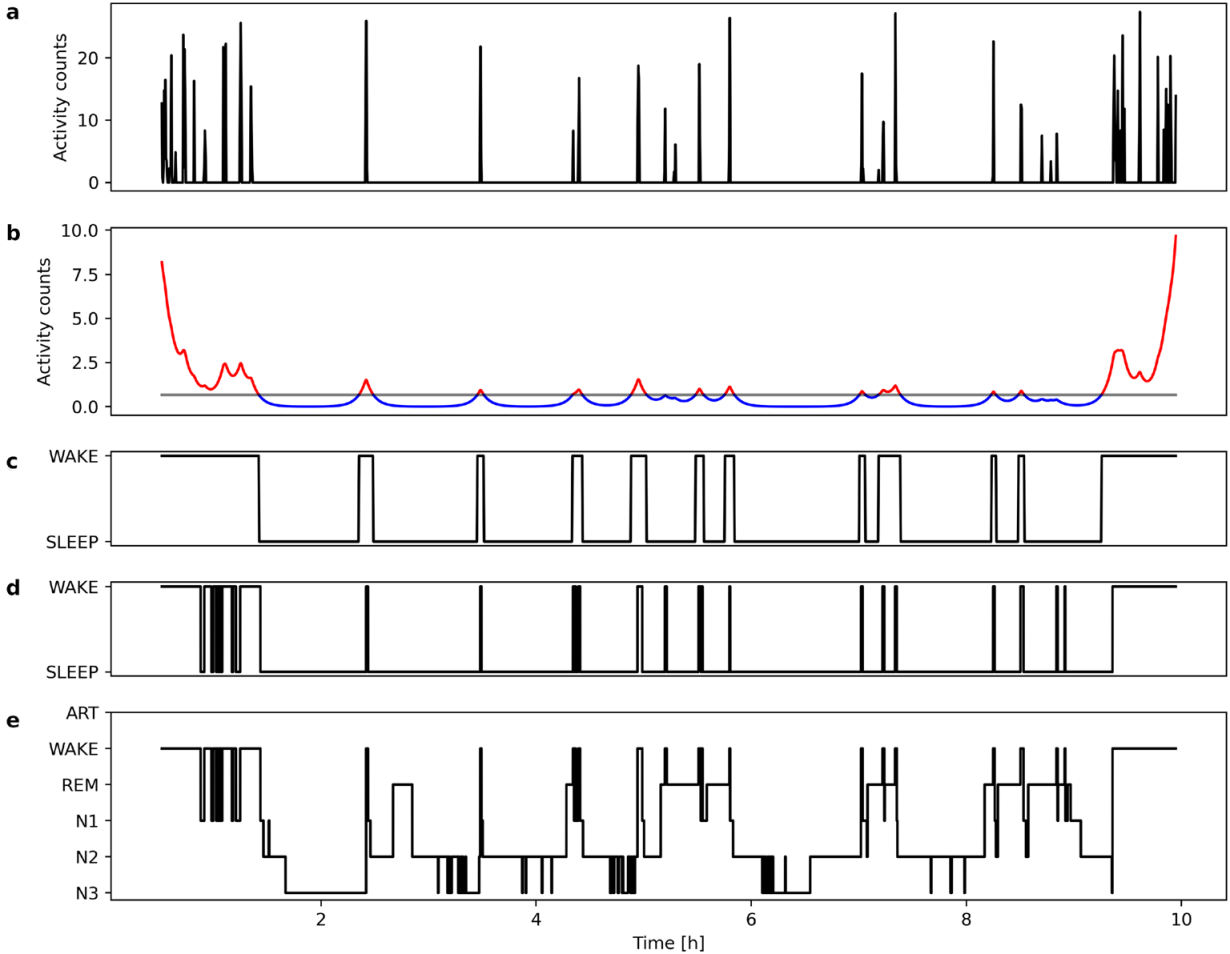
Example signals from an overnight PSG + actigraphy coregistration. **a**: “activity counts”, **b**: result of smoothing (low-pass filtering) of the above. Values above the threshold (red) are interpreted as wake, below (blue)—as sleep. **c**: sleep/wake classification derived from the above thresholding. **d**: sleep/wake classification derived from the hypnogram, presented below in **e**, by concatenating all the sleep stages into “sleep”. Signal in **d** is taken as the ground truth for the evaluation of actigraphic scoring presented in **c**.

Sleep disorders like insomnia, circadian rhythm disorders and obstructive apnea are considered “civilization illnesses” because they have become more prevalent due to modern lifestyles^1–3^. Chronic insomnia affects at least 15% of the population, circadian rhythm disorder about 10% and moderate or severe obstructive apnea about 13–20%^2^. Polysomnography (PSG) is the gold standard for sleep assessment^4–6^. PSG recordings contain electroencephalographic (EEG) and other electrophysiological signals, from which a hypnogram (Fig. 1e) is constructed by trained experts by scoring sleep stages (N1, N2, N3, and REM) in 20- or 30-second epochs^7,8^. It is a relatively expensive and resource-intensive method that requires the use of multiple sensors and electrodes, and it is not feasible to examine large groups of individuals simultaneously.

Actigraphy, a technique that uses simple wrist-worn devices to measure movement intensity, is an alternative to polysomnography for assessing the sleep-wake cycle. It enables longitudinal measurements in natural environments, can be performed over several days to assess habitual sleep behaviour, and is a cost-effective alternative to PSG^9,10^. A recent review quotes promising results regarding the use of actigraphy as a supportive diagnostic and monitoring method for insomnia, narcolepsy, and REM sleep behaviour disorder^11^. Moreover, a systematic review by the American Academy of Sleep Medicine presents evidence supporting the use of actigraphy as an objective tool for assessing sleep/wake patterns and estimating specific sleep parameters in both children and adults across various sleep disorders, especially for identifying sleep continuity in insomnia and for assessing circadian dysrhythmia, and defines recommendations for using it as a clinical tool for evaluating adult and paediatric patients with suspected sleep disorders^6^. The potential of actigraphy as a research technique has been recognized by researchers over the past decades, resulting in a significant increase in its use in scientific research, with over 7,000 scientific articles in the last 10 years^12^.

These applications, based on binary classification of sleep/wake periods, date back to 1980, when Mullaney et al. showed that actigraphic recordings can be used in sleep research by manually scoring the analog signal for sleep/wake periods with a high degree of accuracy compared to polysomnographic (PSG) scoring^13^. In 1982, Webster et al. developed the first automatic method for scoring sleep/wake periods in digitized recordings^14^.

### The first sleep/wake scoring algorithm

In the seminal study by Webster et al., a signal from a piezoelectric activity transducer worn on a watchband was sampled with an effective frequency of 60 Hz, then nonlinearly downsampled to 1-minute intervals, yielding time series *X*(*i*) in the next step convolved with empirically chosen coefficients and thresholded^14^. Assignment of sleep/wake labels to *X*(*i*) was based on the implicit assumptions that (1) activity above a certain threshold corresponds to wake periods and (2) the sleep/wake state should not change too often. The latter assumption requires smoothing of the data before thresholding. Webster et.al. implemented smoothing by means of the convolution defined by the following equation (1)^14^:

1 $$\begin{aligned} & D(i) = 0.025\; \left[ (0.15\; X(i - 4) + 0.15\; X(i - 3) + 0.15\; X (i - 2) + 0.08\; X (i - 1)\right. \nonumber \\ & \left. + 0.21\; X(i) + 0.12\; X(i + 1) + 0.13\; X(i + 2) \right] \end{aligned}$$

Epochs *i* with $D(i)> 1$ were initially classified as “wake”, remaining as “sleep” (Fig. 1d). In the final step, this binary series was subjected to a posterior rescoring, analogous to the rules applied in constructing hypnograms from PSG^15^. All the parameters of both the above-described procedures, including the coefficients in equation (1), as well as formulae for computing “epoch values” and *X*(*i*), were chosen empirically by testing several *a priori* chosen candidates for concordance with PSG.

The approach presented in Webster et al.^14^ set the standard template for the development of new algorithms for sleep/wake scoring in the following decades. It consists of two major stages: (1) downsampling, and (2) smoothing and thresholding^16–21^.

### Downsampling

This stage, often referred to as “data collapsing” or computing “activity counts”, was enforced by hardware limitations of the early actigraphs^22^. Recent technological advances allowed for storing uncompressed data recorded with resolutions up to 100 Hz for several days and replacing the previously used piezoelectric sensors—which returned scalar values nonlinearly related to the acceleration—with microelectromechanical systems (MEMS), recording values of acceleration in units of *g* for the three spatial axes. Nevertheless, some of the contemporary actigraphs still return scalar “activity counts” computed onboard via sometimes undisclosed algorithms^23^. Since unification or standardization of this step is beyond the reach and scope of this study, we concentrate on the second step of smoothing the activity counts. The proposed approach also applies to the modern devices, because researchers often compute “activity counts” offline, to use one of the classical algorithms on such processed data^24–29^.

### Smoothing

Smoothing of the “activity counts” in the second step of the classical algorithms was described in the original papers; formulae similar to equation (1) represent an implicit convolution of the “activity counts” with empirically chosen vectors of coefficients^14,16–19^. For example, in equation (1) that vector would be [0.15, 0.15, 0.15, 0.08, 0.21, 0.12, 0.13]. Based on basic signal processing theory, convolution is a finite impulse response filter (FIR). This seemingly trivial, yet previously unexplored observation was the basis of our previous theoretical work, where we compared the transmittances of the filters implicitly present in the most popular implementations (Cole-Kripke^16^, Webster^14^, UCSD^19^, Scripps Clinic^18^, Sazonov^17^)^30^. Unsurprisingly, all of them turned out to be low-pass filters. Their cutoff frequencies varied from 0.0008 to 0.0015 Hz, with corresponding periods ranging from 10 to 20 minutes^30^. Treating these convolutions as filters brings up some more or less obvious facts:

- coefficients from equation (1) work only for the signal of a particular sampling frequency, used in^14^—the same holds for other propositions^16–19^.
- FIR filters may not be the optimal choice, because the desired properties of a FIR filter may require convolutions with very long sequences, creating border condition issues. Infinite impulse response (IIR) filters offer much better properties for similar filter lengths, at a cost of nonlinear phase shifts, which can be fully compensated by zero-phase filtering in both the forward and reverse directions.

Using the knowledge from the signal processing theory, we may—instead of finding coefficients by trial and error—design filters based on their desired properties, for any sampling frequency.

This study verifies the possibility of constructing a universal smoothing filter which can be used for sleep/wake scoring of any data recorded as “activity counts” after adjusting the threshold. To do so, we use three different datasets processed in distinct ways in order to maximize the universality of the proposed approach. Based on them, we a) optimize a new, universal filter, b) compare 6 different algorithms (including the proposed one) on a uniform dataset, and c) assess their scoring quality in dependence on sampling frequency.

## Methods

### Experimental data

Concordance of different PSG scoring algorithms with PSG was evaluated on three different datasets—two available online and one recorded for this study. Two of them were recorded in a laboratory setting, and one at the subject’s home, yielding a total of 1635 overnight recordings of polysomnographic (PSG) and actigraphic signals used after rejecting faulty data. PSG was scored by experts, and stages N1, N2, N3 and REM were combined and labeled “sleep”, while wake was preserved as “wake”. Epochs scored as unidentified (or as artifacts) were not taken into account when calculating metrics or optimizing the algorithm.

Dataset 1 and Dataset 2 contained raw acceleration values from the *GeneActiv Original* devices (ActivInsights LTD), which were offline converted to 1-dimensional “activity counts”, in both cases using two distinct algorithms: Activity Index^31^, or MIMS^32^; details of their operation can be found in the Supplementary Methods. Actigraphic and PSG data were synchronized prior to epoching, ensuring the best alignment. Actigraphic data in Dataset 3 are limited to “activity counts” and were synchronized after collapsing data into epochs. Synchronization details can be found in the Supplementary Methods.

**Dataset 1** Data collection was a part of a study at the University of Warsaw, approved by the Rector’s Committee for the Ethics of Research Involving Human Participants at the University of Warsaw, Poland (approval number 224/2023). The study was conducted according to the guidelines provided by the Committee, and all participants signed an informed consent form prior to data registration. The dataset yields 43 overnight recordings of healthy adults (PSG consisting of 21 EEG electrodes, chin EMG, ECG and EOG derivations). Out of them, 9 recordings were rejected prior to analysis due to technical issues. For all the healthy volunteers (mean age $22.1 \pm 3.5$ years, 21 males and 22 females), this was the first night in the laboratory setting, without an adaptation night, which is reflected in relatively low quality of sleep. The actigraphs were placed on the non-dominant wrists (6 participants were left-handed). PSG was scored in 20 s epochs by an experienced expert based on AASM criteria; the sampling frequency of the actigraphs was 75 Hz^33^.

**Dataset 2** Eighty recordings from the EESM19 dataset by Mikkelsen et al. were used^34^. These were performed at the homes of the 20 participants (mean age 25.9 years, ranging from 22 to 36 years, with 13 females and 7 males), each of whom was recorded 4 times. This dataset consisted of coregistered actigraphic data and PSG (without ECG). PSG was scored in 30 s epochs by an experienced expert according to AASM rules^8^; sampling frequency of the actigraphs was 100 Hz.

**Dataset 3** The MESA dataset contains 1749 recordings with coregistration of actigraphy and PSG collected from individuals with a mean age of $68.4 \pm 9.1$ years, out of which 53.6% were female^35,36^. A total of 1,521 recordings were used, as some values in the actigraphic data were missing during the period in which PSG was collected in each of the remaining 228 recordings. PSG was scored in 30 s epochs by multiple experts (one per recording) according to the rules outlined in the MESA documentation, and actigraphic recordings contained only “activity counts”, returned by the proprietary firmware of *Actiwatch Spectrum* actigraphs (Philips Respironics) in 30 s epochs^35,36^.

### Optimizing the universal filter

A filter applicable to data collected with different sampling rates cannot be given in terms of fixed coefficients like equation (1). In order to find a possibly general solution, we first chose an IIR elliptic filter as the type of filter. Out of the four common IIR filter types, this allows for the most extreme configuration while preserving numerical stability.

**Fig. 2:**
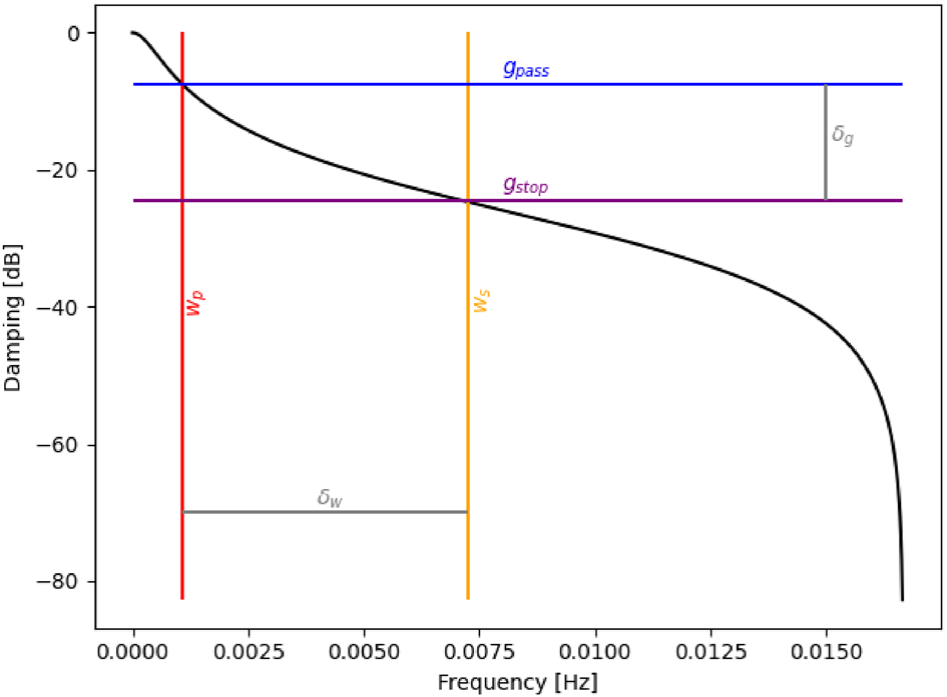
An example transmittance of a low-pass filter. Vertical red line marks the edge frequency of the pass-band, vertical orange line marks the edge frequency of the stop-band, purple horizontal line marks minimal attenuation in the stop-band, and blue horizontal line—maximal attenuation in the pass-band. Grey lines mark the widths of the transition band in frequency and damping.

In the second step, we chose the ranges of its parameters:

1. Frequency at which pass-band stops $w_p \in [\frac{1}{7500}, \frac{1}{400}]$ Hz;
2. Delta frequency $\delta _w$ such that $w_s = w_p + \delta _w$ and $\delta _w \in [\frac{1}{1000}, \frac{1}{100}]$ Hz;
3. Maximal damping in pass-band $g_\text {pass} \in [-8, -\frac{1}{100}]$ dB;
4. Delta damping $\delta _g$ such that minimal damping in stop-band $g_\text {stop} = g_\text {pass} - \delta _g$ and $\delta _g \in [1, 40]$ dB;

These parameters are visualized for convenience in Fig. 2. Optimizing $\delta _w$ instead of $w_s$ ensured that only low-pass filters will be found as solutions, as by definition, low-pass filters must satisfy $w_s> w_p$. By definition $g_\text {pass}> g_\text {stop}$ has to be satisfied for every filter, hence optimizing $\delta _g$ instead of $g_\text {stop}$. Chosen boundaries on $w_p$ ensure a wide range of pass-band widths, including cutoffs of classic algorithms; boundaries on $\delta _w$ cover all cases from a very narrow transition band up to $w_s$ near the Nyquist frequency. Also, the boundaries of $g_\text {pass}$ and $\delta _g$ allowed searching a very wide range of damping values—including damping of classic algorithms.

Values of these parameters—together with the value of the threshold separating sleep from wake, taken from the interval $[\frac{1}{1000}, 10]$—were optimized for concordance with the sleep/wake scorings derived from PSG, using the Nelder-Mead algorithm as implemented in SciPy^37^.

The optimization was done on the whole Datasets 1 and 2. In both cases, the process was performed twice—using data epoched with Activity Index, and with MIMS algorithms^31,32^. These open source algorithms are designed to output “activity counts”, with properties similar to those returned by old piezoelectric devices. They differ, however, in the mode of operation and therefore in the properties of output data. Details about their definitions can be found in Supplementary Methods. By using both of these algorithms, we simulate a case in which similar data (e.g. from one population) is collected using different devices with proprietary “activity counts” algorithms builtin.

On each of the 4 sets described (2 transformations of 2 other datasets), the optimization process was repeated 500 times, with random initial values of all parameters, in order to minimize the influence of local maxima on the selection of the best filter. Out of all filters fitted during 500 iterations, the one achieving the highest correlation between actigraphy and PSG was selected for use in further analyses. We obtained 4 filters fitted on different (although not fully independent) data.

### Statistics reflecting the performance

Sleep/wake scoring yields a substantially unbalanced problem because of the natural prevalence of sleep during the night. For example, on a sleep recording containing 10% of wake time, a bogus algorithm marking all the epochs as sleep would achieve 100% sensitivity, 0% specificity, and 90% accuracy. Such statistics are used to report the concordance with PSG in a large number of actigraphic studies, so for the sake of comparability, we quote them also in this study^16–19,24,27–29,38,39,42^. Nevertheless, we advocate using Mathew’s Correlation Coefficient (MCC) as an alternative robust against unbalanced classes in data—for the above example, it would correctly return 0^43^. Moreover, in the case of binary classification, it is equivalent to Pearson’s correlation coefficient. Cohen’s $\kappa$ is also robust against class imbalance, but its interpretation is much less intuitive^44,45^. Detailed description of all used metrics can be found in the Supplementary Methods.

## Results

### The universal filter

**Fig. 3:**
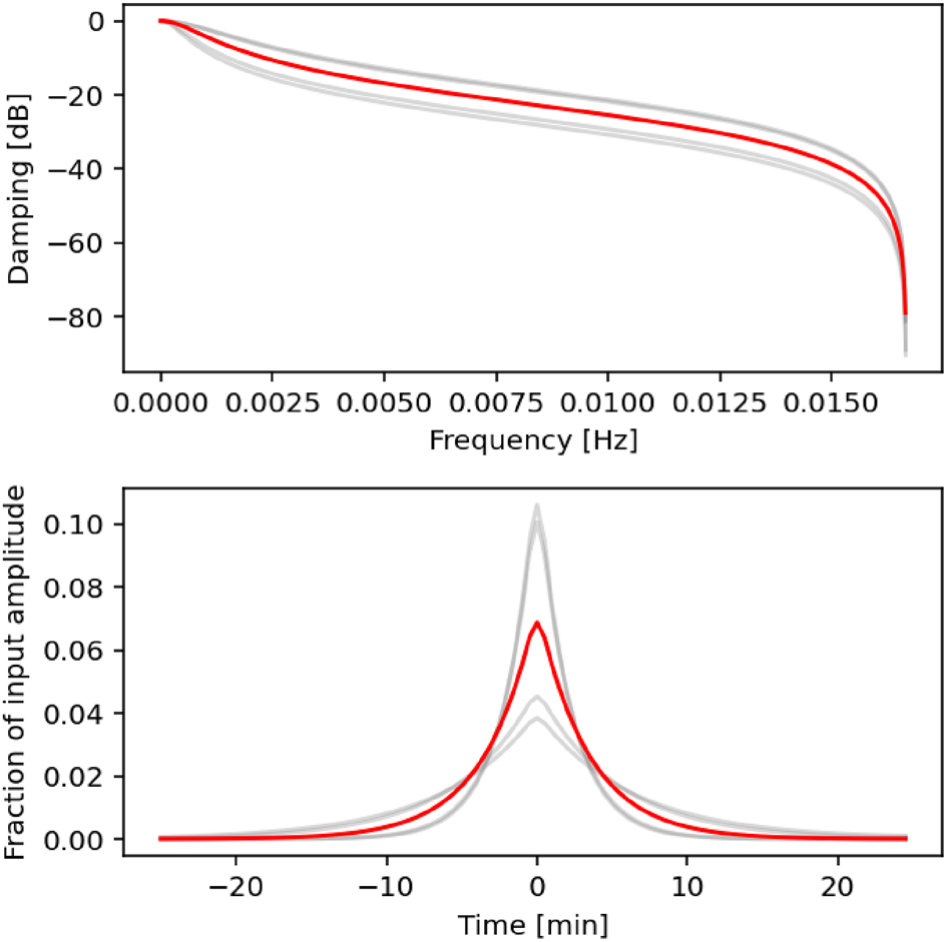
Grey lines—transmittances (upper panel) and impulse responses (lower panel) of filters fitted to the four non-overlapping datasets. Red lines depict properties of the average (universal) filter.

In the first step, transmittances and impulse responses of the 4 obtained filters were compared. Grey lines in Fig. 3 present these properties. Since all these filters are represented by very similar transmittances and impulse responses, their parameters were averaged, and these averages were taken as tentative parameters of the universal filter. Its transmittance and impulse response are plotted in the same Fig. 3 in red. Its parameters, shown in Table 1, reveal relatively large standard deviations; however, this does not necessarily mean that these filters, fitted to considerably different datasets, were indeed substantially different. Similar transmittances and impulse responses, as those in Fig. 3, can be achieved with different combinations of the filter parameters. For example, increasing $w_s$ with a proportional decrease in $g_\text {stop}$ may not lead to a drastic change in transmittance shape—at least as long as the filter stability is preserved. Based on this assumption, we proceeded towards comparing the performance of the universal filter with the traditional algorithms.

**Table 1:** Parameters defining the universal filter, together with their standard deviations.

pass-band edge [Hz]	stop-band edge [Hz]	pass-band damping [dB]	stop-band damping [dB]
$8.10 \cdot 10^{-4} \pm 3.60 \cdot 10^{-4}$	$8.44 \cdot 10^{-3} \pm 2.30 \cdot 10^{-3}$	$3.17 \pm 2.56$	$14.86 \pm 5.89$

### Performance of scoring algorithms

We implemented five of the widely used algorithms proposed for actigraphic sleep/wake scoring in previous studies: Webster^14^, Cole-Kripke^16^, Sazonov^17^, UCSD^19^, and Scripps Clinic^18^. Taking the hereby proposed universal filter as the sixth, we evaluate their performance on the Dataset 3 (MESA) because it is the largest dataset available, and has already been used in algorithms evaluation study^38^. For each algorithm, the same 10% of data (152 recordings) was used to fit the threshold. The remaining 90% (1369 recordings) were scored independently by each algorithm.

**Fig. 4:**
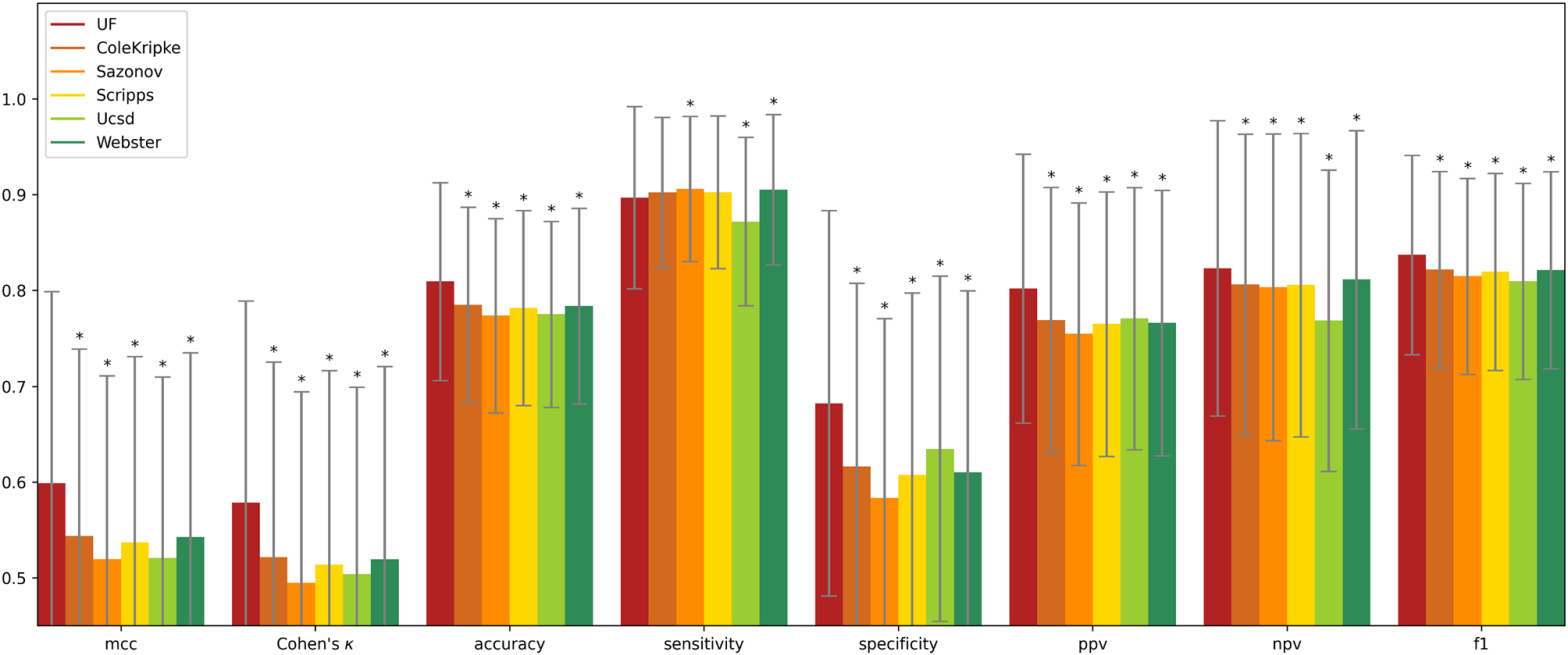
Mean values of several measures assessing concordance with PSG scorings, together with standard deviations. Asterisks mark values, which are significantly different than corresponding values for the universal filter, as tested with a paired two-sided Wilcoxon test at $p <0.001$ with Bonferroni correction.

As presented in Table 2 and Fig. 4, in all commonly reported measures except for sensitivity, the universal filter performs significantly better than previously proposed algorithms, yielding significantly higher concordances with $p<0.001$ from a two-sided paired Wilcoxon test with Bonferroni correction. Table 3 presents estimates of the effect sizes of differences between metrics distributions when comparing the universal filter to other algorithms. Effect size is reported by z-scored statistic from the Wilcoxon test divided by the square root of the number of data points (twice the sample size in the case of the Wilcoxon test)^46^.

For reference, Supplementary Table S4 also quotes the results achieved by classic algorithms when used on the same MESA testing set with their default thresholds. While unbalanced metrics vary extensively, MCC and Cohen’s $\kappa$ are unsurprisingly lower than after threshold calibration, highlighting the need for adjusting the algorithm for a given device and collapsing method pair.

**Table 2:** Mean values of statistics assessing concordance with PSG scorings, together with standard deviations. Bold font marks values, which are significantly different than corresponding values for the universal filter, as tested with a paired two-sided Wilcoxon test at $p <0.001$ with Bonferroni correction.

	MCC	Cohen’s $\kappa$	accuracy	sensitivity	specificity	ppv	npv	f1
ColeKripke	$\mathbf {0.54 \pm 0.20}$	$\mathbf {0.52 \pm 0.20}$	$\mathbf {0.78 \pm 0.10}$	$0.90 \pm 0.08$	$\mathbf {0.62 \pm 0.19}$	$\mathbf {0.77 \pm 0.14}$	$\mathbf {0.81 \pm 0.16}$	$\mathbf {0.82 \pm 0.10}$
Sazonov	$\mathbf {0.52 \pm 0.19}$	$\mathbf {0.49 \pm 0.20}$	$\mathbf {0.77 \pm 0.10}$	$\mathbf {0.91 \pm 0.08}$	$\mathbf {0.58 \pm 0.19}$	$\mathbf {0.75 \pm 0.14}$	$\mathbf {0.80 \pm 0.16}$	$\mathbf {0.81 \pm 0.10}$
Scripps	$\mathbf {0.54 \pm 0.19}$	$\mathbf {0.51 \pm 0.20}$	$\mathbf {0.78 \pm 0.10}$	$0.90 \pm 0.08$	$\mathbf {0.61 \pm 0.19}$	$\mathbf {0.76 \pm 0.14}$	$\mathbf {0.81 \pm 0.16}$	$\mathbf {0.82 \pm 0.10}$
Ucsd	$\mathbf {0.52 \pm 0.19}$	$\mathbf {0.50 \pm 0.20}$	$\mathbf {0.78 \pm 0.10}$	$\mathbf {0.87 \pm 0.09}$	$\mathbf {0.63 \pm 0.18}$	$\mathbf {0.77 \pm 0.14}$	$\mathbf {0.77 \pm 0.16}$	$\mathbf {0.81 \pm 0.10}$
Webster	$\mathbf {0.54 \pm 0.19}$	$\mathbf {0.52 \pm 0.20}$	$\mathbf {0.78 \pm 0.10}$	$\mathbf {0.91 \pm 0.08}$	$\mathbf {0.61 \pm 0.19}$	$\mathbf {0.77 \pm 0.14}$	$\mathbf {0.81 \pm 0.16}$	$\mathbf {0.82 \pm 0.10}$
Universal Filter	$0.60 \pm 0.20$	$0.58 \pm 0.21$	$0.81 \pm 0.10$	$0.90 \pm 0.10$	$0.68 \pm 0.20$	$0.80 \pm 0.14$	$0.82 \pm 0.15$	$0.84 \pm 0.10$

**Table 3:** Effect sizes measured by z-scored statistic from the Wilcoxon test divided by the square root of the number of data points, testing for the difference between values of a given metric for a given algorithm, and the corresponding value for the universal filter.

	MCC	Cohen’s $\kappa$	accuracy	sensitivity	specificity	ppv	npv	f1
ColeKripke	0.49	0.50	0.47	0.06	0.57	0.57	0.19	0.40
Sazonov	0.56	0.57	0.54	0.13	0.60	0.60	0.21	0.47
Scripps	0.52	0.53	0.51	0.08	0.59	0.59	0.21	0.44
Ucsd	0.53	0.52	0.51	0.35	0.44	0.52	0.45	0.50
Webster	0.52	0.52	0.50	0.13	0.59	0.59	0.14	0.42

The above analysis was performed according to the cross-validation rules, that is both the filter parameters as well as thresholds were optimized on the subset of data which was not used for computing the performance metrics. However, from the point of view of practical application of this filter to a completely new dataset, it’s tempting to optimize the filter on all the available data. This approach is presented in the Supplementary Material, where Supplementary Table S2 lists the parameters of the filter fitted to the same 4 datasets described in the Methods section, plus 5 randomly selected subsets of the MESA dataset (we do not use the complete MESA dataset to keep the training data balanced). Both the parameters of the filter fitted in such way (Supplementary Table S2) and its performance (Supplementary tables S3 and S4) do not differ significantly from the above results obtained with strict cross-validation, which further supports the claim regarding high universality of the proposed solution.

### Sampling frequency independence

**Fig. 5:**
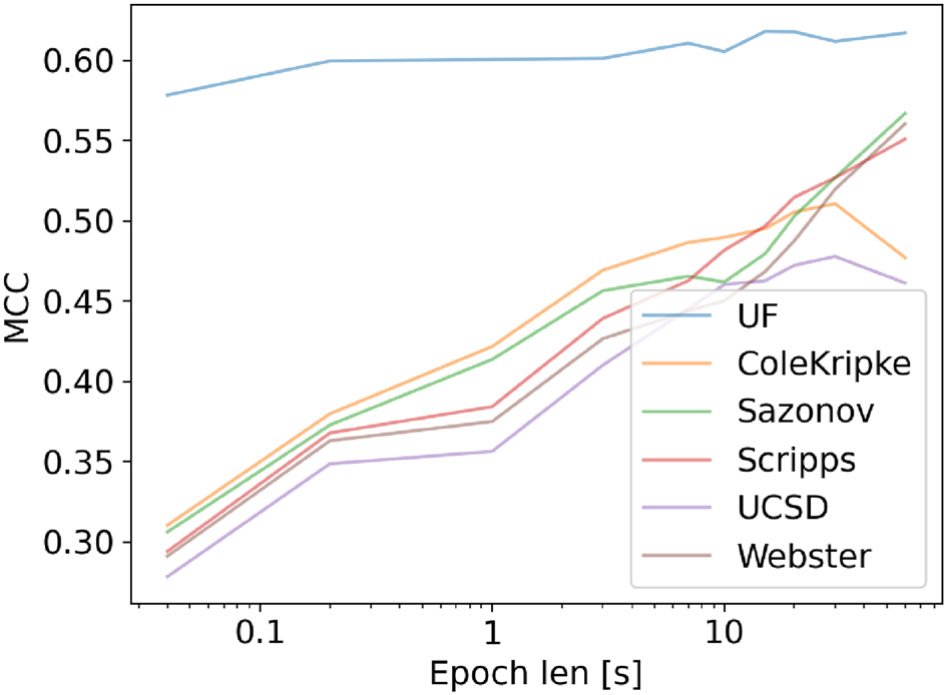
Mean correlation between actigraphic and PSG scorings for different epoch lengths, estimated for the previously published approaches and the proposed universal filter (UF).

Contrary to the proposed approach, each of the previously published algorithms was designed for a specific sampling frequency. In spite of that, in some studies they were used on data collapsed into epochs of different lengths^24–28,38–40,42^. While such a situation may be formally treated as a methodological error, epoch lengths remained in the range of 20–60 seconds, where the classification quality does not degrade drastically, as can be seen in Fig. 5. Another issue relates to the fact that the downsampling procedures (“data collapsing”) in actigraphy are nonlinear, so the invariability of the filter’s properties does not automatically guarantee optimal performance for all the epoch lengths. To verify and illustrate these issues, Fig. 5 presents the quality of classification achieved by different algorithms over a wide range of epoch lengths—details of their estimation can be found in Supplementary Methods.

## Discussion

Optimal filters for sleep/wake classification, estimated for each of the four subsets of the available data, were described by similar transmittances and impulse responses. This encouraged us to construct a universal filter from the average of their parameters. This universal filter outperformed five of the previously proposed algorithms widely used in actigraphic research—with a noteworthy improvement in specificity, previously deteriorated by the problems with detecting wake periods from actigraphic recordings^4,10,47,48^. Contrary to the previous approaches, the universal filter is also fully independent of the sampling frequency of the input data, both in terms of the filter properties and resulting sleep/wake classification.

However, the proposed approach is not without limitations. For example, it was both trained and validated using only data from healthy participants. While low sleep quality in Dataset 1 increases variability in the data to some extent, the sample of patients with sleep disorders would certainly present different sleep properties. It is not obvious how using such a sample would influence classification quality. Most important limitations, however, stem from the usage of “activity counts” as an input.

Contemporary devices can record exact values of acceleration across 3 spatial dimensions with high temporal resolution, typically going up to 6000 samples per minute. Compared to a single scalar representing each minute, such a signal carries much more information, which potentially could improve the quality of sleep/wake scoring. Examples of algorithms working directly on such data are, e.g. van Hees algorithm^41^ or Hidden Markov Models used by Trevenen et al.^49^. The approach to actigraphic sleep/wake scoring discussed in this study can be, to an extent, seen as a relic of past technology—the future of actigraphy seems to pivot towards direct analysis of raw, 3-axial data by more sophisticated methods^38,49,50^. Nevertheless, researchers still rely extensively on these traditional algorithms, which are believed to be robust, verified, and compatible with older studies^51^. Unfortunately, as presented in several comparative studies, not only do different algorithms yield different (albeit generally similar) results on the same data, but the same may also occur for different implementations of the same algorithm, which leaves room for improvement in terms of unification and informed design of these algorithms based on the robust theory of signal processing^28,51–54^.

Another source of scoring errors may stem from the non-standard and sometimes proprietary ways of computing the “activity counts”. According to Neishabouri et al.^22^, *the obscurity of the count algorithms has also led to the common misconception that “counts”is a universal unit of measurement that is the same across devices, where [...] variations in parameters [...] can lead to vastly different counts*—a conclusion supported by several studies^32,55,56^. Since in many cases we have no influence on this step, an explicit separation of the filtering and thresholding steps, supplemented by the abovementioned informed design, seems to be the only way to reduce bias.

In light of the presented results, the proposed universal filter approach can be treated as a device-independent solution, while the sleep/wake threshold should be calibrated for a given pair of device/collapsing method—a limitation inherent to any approach based on “activity counts”. Overall, despite the availability of MEMS-based 3-axial devices, different legacy algorithms are still extensively used^28,38,40,48^. The proposed approach maintains their general *modus operandi*, giving significantly superior results, and paves the way for designing future algorithms within the mathematical framework of signal processing.

## Supplementary Information

Supplementary Information.

## Data Availability

Dataset 1 collected during the study is freely available from https://danebadawcze.uw.edu.pl/dataset.xhtml?persistentId=doi:10.58132/DCQYO1. Python code needed to fully replicate described analysis will be made public under GPL-3 license together with the release of Python package for actigraphic analysis, on which we are currently working. Until then, the code used for computations in this study is available on request from the first author.
