# Supplementary material for: Universal approach to actigraphic sleep/wake scoring, verified against 5 classic algorithms on 3 datasets
Source: Sci Rep. 2026 Apr 17;16:17878. doi: 10.1038/s41598-026-45568-0 (PMC13249932; doi:10.1038/s41598-026-45568-0)
Supplement: Supplementary file 1 — Supplementary Information. [file 41598_2026_45568_MOESM1_ESM.pdf]

Supplementary information for the article
Universal approach to actigraphic sleep/wake scoring, verified against 5 classic algorithms on 3 datasets
by Biegański et.al., Scientific Reports 2026

Supplementary methods

Used statistics

For the sake of clarity let us consider actigraphic algorithms as sleep detectors. Then, the confusion matrix for sleep/wake classification problem can be formulated as in Table S1.

		Polysomnography	
		Sleep	Wake
Actigraphy	Sleep	TP	FP
	Wake	FN	TN

Table S1: Confusion matrix for actigraphic sleep/wake classification problem (i.e. definitions of notions used in metric formulations). TP: true positive; TN: true negative; FP: false positive; FN: false negative.

Using these notions we can define measures, which are used throughout the text. The list include:

- Accuracy: ACC $= \frac{TP+TN}{TP+TF+FN+FP}$
- Specificity: SPE $= \frac{TN}{TN+FP}$
- Sensitivity: SEN $= \frac{TP}{TP+FN}$
- Positive predictive value: PPV $= \frac{TP}{TP+FP}$
- Negative predictive value: NPV $= \frac{TN}{TN+FN}$
- F1 score: F1 $= \frac{2TP}{2TP+FP+FN}$
- Cohen's Kappa [2]: $\kappa = \frac{2\times(TP\times TN-FN\times FP)}{(TP+FP)\times(FP+TN)+(TP+FN)\times(FN+TN)}$
- Mathew's Correlation Coefficient [4]: MCC $= \frac{TP\times TN-FP\times FN}{\sqrt{(TP+FP)(TP+FN)(TN+FP)(TN+FN)}}$

Synchronization of PSG scoring with actigraphy

The following procedure was used to synchronized actigraphy with sleep/wake scorings obtained from PSG staging for datasets 1 and 2:

1. The PSG sleep/wake scorings were upsampled to the sampling frequency of actigraphic data by repeating each point necessary number of times.
2. Raw actigraphic data was synchronized with such upsampled PSG staging by minimizing the distance between timestamps of actigraphic samples and upsampled PSG staging samples—thus resulting in a synchronization with an error equal to at most $1/f_s$ s, where f_s is sampling frequency of actigraphic data.
3. The actigraphic data was then converted into "activity counts" (see below) of desired length (e.g. 30 seconds), while PSG staging was collapsed back into 30-s long epochs using following rule: if at least 80% of points in the 30-s window were labeled wake, then the window was labeled wake; sleep otherwise. Coefficient 80% was chosen empirically and proved to change the resulting sleep/wake classification to a small extent in comparison to the original one (prior to synchronization).

Collapsing into activity counts

Activity Index

Activity Index by Bai et al. [1] is designed only for 3-axial, MEMS-based devices and can be broken down into the following steps:

1. Signal is divided into windows of desired length in seconds (e.g. 30 or 60), let i-th window H_i.
2. Let the variance of stationary actigraph (i.e. variance of the digital converter noise) σ_s^2.
3. For each axis in $a \in \{x, y, z\}$ compute the variance of the signal in window H_i, $\sigma_a^2(H_i)$
4. Then, the Activity Index of epoch H_i is defined as:

$$\mathrm{AI}_i = \sqrt{\max\left(\sum_{a \in \{x,y,z\}} \frac{\sigma_a^2(H_i) - \sigma_s^2}{3\sigma_s^2}, 0\right)} \tag{1}$$

MIMS

MIMS by John et al. [3] is in principle usable on MEMS-based devices with any number of axes, below we consider a case with 3. The algorithm is described by the following steps:

1. Each axis is resampled to sampling frequency of 100 Hz.
2. Fragments, in which the signal is maxed out (i.e. the acceleration was higher, than the dynamic range of the device) are found, and acceleration values are interpolated using cubic splines.
3. Each axis is bandpass filtered using fourth-order Butterworth filter with pass-band in 0.2-5 Hz. Let the signal after these operations $a \in \{x, y, z\}$.
4. Signal is divided into windows of desired length in seconds (e.g. 30 or 60), let i-th window H_i.
5. Then, the MIMS value of epoch H_i is defined as, using trapezoid integral as a numerical approximation:

$$\mathrm{MIMS}_i = \sum_{a \in \{x,y,z\}} \int_{H_i} |a| \tag{2}$$

6. In the final step, values less than 0.001 are replaced by 0.

Sampling frequency independence

To assess the dependence of scoring quality on epoch length we've used combined datasets 1 and 2, as the data in MESA are available only already epoched. From the combined datasets (yielding 114 recordings in total), randomly selected 20% of recordings were used as a training set on which a threshold was fitted for each algorithm separately (by the means of maximizing MCC between actigraphic and PSG scorings), while the remaining 80% served as a test set on which mean MCC was computed and recorded for each considered epoch length and algorithm.

On the testing set mean correlation between PSG and actigraphic scoring was calculated for different epoch lengths in the range ranging from 1/25 second to 60 seconds. Data was collapsed into epochs of different lengths using the Activity Index method [1]; the threshold was fitted separately for each epoch length and for each algorithm.

For epochs shorter than the original epoch length, PSG sleep/wake classification was resampled by repeating the corresponding label an adequate number of times (e.g. one 30 second-long sleep epoch is treated as three 10 second-long sleep epochs, etc.). In the case of 60 second epoching, we classified a new epoch as wake if all points in the original epochs in the 60 second-long window were classified as wake.

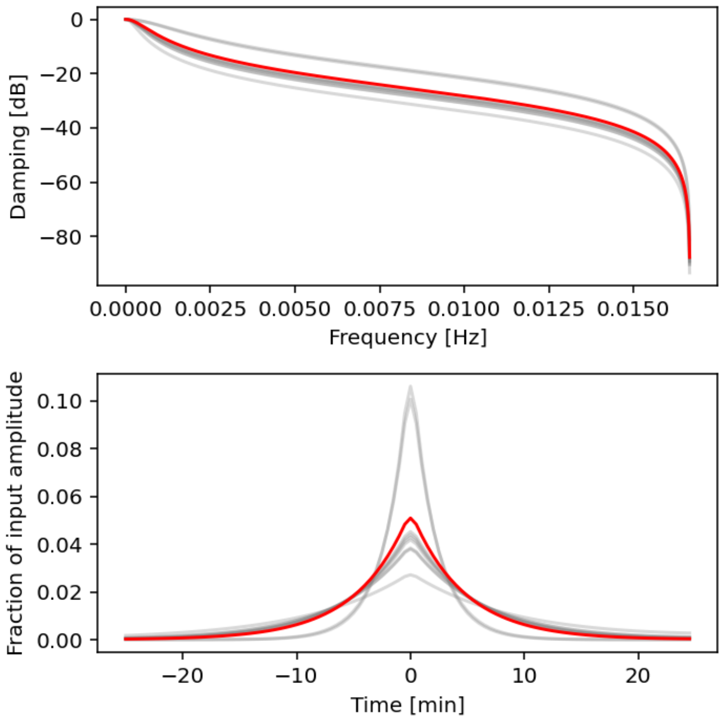

Figure S1: Transmittance (upper panel) and impulse response (lower panel) of filter obtained by averaging parameters of 9 filters fitted on Datasets 1, 2 and 3. In red the average filer. In light gray 9 filters described in the main text. Impulse was present at the moment $t = 0$.

pass-band edge [Hz]	stop-band edge [Hz]	pass-band damping [dB]	stop-band damping [dB]
$6.41 \cdot 10^{-4} \pm 3.17 \cdot 10^{-4}$	$6.81 \cdot 10^{-3} \pm 3.05 \cdot 10^{-3}$	3.57 ± 2.23	14.59 ± 5.84

Table S2: Parameters defining the universal filter averaged from 9 filters, together with their standard deviations.

Supplementary results

Averaging 9 filters

As described in the Section Performance of scoring algorithms of the main text, we've also conducted analysis averaging 9 filters not fitted on MESA to ensure no overfitting. We've used 4 datasets described in the main text, as well as 5 randomly selected subsets of the MESA dataset, each with 114 recordings. Figure S1 presents its properties and Table S2 the mean parameters together with standard deviations.

We've also conducted comparison with classic algorithms on MESA dataset in the same way, as described in the main text—10% of data used for threshold fitting for all algorithms and 90% used to calculate values of the metrics. Tables S3 and S4 present results of comparison between such a filter, and classic algorithms. Just like in case of the main analysis, significance was tested with one-sided paired Wilcoxon test at $p < 0.001$. and effect size was estimated via the coefficient described in the main text. Mean values of metrics are identical, as in case of the universal filter, with the only difference being 0.01 higher npv. Effect sizes are also similar.

Classic algorithms with default threshold

For a reference, the same recordings from the MESA dataset that were used as a testing set were used to assess performance of all five classic algorithms with a default thresholds. While the metrics vary quite extensively in comparison to the fitted thresholds, the unbiased metrics (MCC and Cohen's κ are in all cases smaller (or equal, only in case of UCSD algorithm), than after fitting a threshold, meaning the real, overall agreement without threshold fitting is worse (or more-less equal in case of UCSD), than with fitting.

	MCC	Cohen's κ	accuracy	sensitivity	specificity	PPV	NPV	F1
ColeKripke	$\mathbf{0.54 \pm 0.20}$	$\mathbf{0.52 \pm 0.20}$	$\mathbf{0.78 \pm 0.10}$	$\mathbf{0.90 \pm 0.08}$	$\mathbf{0.62 \pm 0.19}$	$\mathbf{0.77 \pm 0.14}$	$\mathbf{0.81 \pm 0.16}$	$\mathbf{0.82 \pm 0.10}$
Sazonov	$\mathbf{0.52 \pm 0.19}$	$\mathbf{0.49 \pm 0.20}$	$\mathbf{0.77 \pm 0.10}$	0.91 ± 0.08	$\mathbf{0.58 \pm 0.19}$	$\mathbf{0.75 \pm 0.14}$	$\mathbf{0.80 \pm 0.16}$	$\mathbf{0.81 \pm 0.10}$
Scripps	$\mathbf{0.54 \pm 0.19}$	$\mathbf{0.51 \pm 0.20}$	$\mathbf{0.78 \pm 0.10}$	0.90 ± 0.08	$\mathbf{0.61 \pm 0.19}$	$\mathbf{0.76 \pm 0.14}$	$\mathbf{0.81 \pm 0.16}$	$\mathbf{0.82 \pm 0.10}$
Ucsd	$\mathbf{0.52 \pm 0.19}$	$\mathbf{0.50 \pm 0.20}$	$\mathbf{0.78 \pm 0.10}$	$\mathbf{0.87 \pm 0.09}$	$\mathbf{0.63 \pm 0.18}$	$\mathbf{0.77 \pm 0.14}$	$\mathbf{0.77 \pm 0.16}$	$\mathbf{0.81 \pm 0.10}$
Webster	$\mathbf{0.54 \pm 0.19}$	$\mathbf{0.52 \pm 0.20}$	$\mathbf{0.78 \pm 0.10}$	0.91 ± 0.08	$\mathbf{0.61 \pm 0.19}$	$\mathbf{0.77 \pm 0.14}$	$\mathbf{0.81 \pm 0.16}$	$\mathbf{0.82 \pm 0.10}$
Unified5	0.60 ± 0.20	0.58 ± 0.21	0.81 ± 0.10	0.90 ± 0.09	0.68 ± 0.21	0.80 ± 0.14	0.83 ± 0.15	0.84 ± 0.10

Table S3: Metrics values for classic algorithms and for averaged 9 filters. In bold are marked values, which are significantly different, than corresponding values for averaged filter (with $p < 0.001$).

	MCC	Cohen's κ	accuracy	sensitivity	specificity	ppv	npv	f1
ColeKripke	0.47	0.47	0.46	0.08	0.51	0.53	0.26	0.41
Sazonov	0.54	0.55	0.54	0.02	0.59	0.59	0.27	0.48
Scripps	0.50	0.51	0.50	0.08	0.55	0.56	0.27	0.44
Ucsd	0.51	0.49	0.50	0.39	0.36	0.47	0.46	0.49
Webster	0.49	0.49	0.48	0.03	0.53	0.55	0.21	0.43

Table S4: Effect sizes for differences between metrics for given algorithms and averaged 9 filters, as measured by z-scored T-statistic divided by square of number of observations.

	mcc	Cohen's κ	accuracy	sensitivity	specificity	ppv	npv	f1
ColeKripke	0.41 ± 0.19	0.36 ± 0.20	0.67 ± 0.12	0.52 ± 0.20	0.88 ± 0.13	0.86 ± 0.15	0.57 ± 0.16	0.62 ± 0.19
Sazonov	0.49 ± 0.19	0.47 ± 0.19	0.75 ± 0.09	0.75 ± 0.13	0.74 ± 0.16	0.81 ± 0.13	0.68 ± 0.16	0.77 ± 0.11
Scripps	0.53 ± 0.19	0.51 ± 0.20	0.78 ± 0.10	0.82 ± 0.11	0.71 ± 0.18	0.80 ± 0.14	0.73 ± 0.16	0.80 ± 0.11
Ucsd	0.52 ± 0.19	0.50 ± 0.20	0.77 ± 0.10	0.87 ± 0.09	0.63 ± 0.18	0.77 ± 0.14	0.77 ± 0.16	0.81 ± 0.10
Webster	0.49 ± 0.19	0.47 ± 0.19	0.74 ± 0.10	0.69 ± 0.15	0.81 ± 0.15	0.84 ± 0.14	0.65 ± 0.16	0.74 ± 0.12

Table S5: Metrics of all considered classic algorithms, when used to score data from the same MESA subset, which was used for testing in the main Results.

References

[1] Bai, J., Di, C., Xiao, L., Evenson, K. R., LaCroix, A. Z., Crainiceanu, C. M., & Buchner, D. M. (2016). An activity index for raw accelerometry data and its comparison with other activity metrics. PloS one, 11(8), e0160644.

[2] Cohen, J. (1960). A coefficient of agreement for nominal scales. Educational and psychological measurement, 20(1), 37-46.

[3] John, D., Tang, Q., Albinali, F., & Intille, S. (2019). An open-source monitor-independent movement summary for accelerometer data processing. Journal for the Measurement of Physical Behaviour, 2(4), 268-281.

[4] Matthews, B. W. (1975). Comparison of the predicted and observed secondary structure of T4 phage lysozyme. Biochimica et Biophysica Acta (BBA)-Protein Structure, 405(2), 442-451.
